# Latent transition analysis in organizational psychology: A simplified “how to” guide by using an applied example

**DOI:** 10.3389/fpsyg.2022.977378

**Published:** 2022-11-08

**Authors:** Jetmir Zyberaj, Cafer Bakaç, Sebastian Seibel

**Affiliations:** ^1^Work and Organizational Psychology Group, Department of Psychology, University of Bamberg, Bamberg, Germany; ^2^TUM School of Management, Technical University of Munich, Munich, Germany; ^3^Work and Organizational Psychology Group, Department of Psychology, University of Wuerzburg, Wuerzburg, Germany

**Keywords:** latent transition analysis, person-centered approaches, “how to” guide, psychological capital, leader-member exchange

## Abstract

Demands for more robust designs in organizational research have led to a steady increase in the number of longitudinal studies in organizational psychology (OP) journals. Similarly, the number and ways to analyze longitudinal data have also increased. In this paper, we adopt a relatively new and promising approach to help researchers analyze their longitudinal data in OP, namely latent transition analysis (LTA). We present a simplified guideline on LTA and discuss its role for OP researchers. Moreover, we demonstrate how organizational scholars can use this method with a practical example. In this example, we investigate (a) if there are qualitatively distinct subgroups of employees based on particular patterns of psychological capital (PsyCap) dimensions (i.e., efficacy, hope, resilience, and optimism), (b) if employees stay in these subgroups or transition to other groups over time, and finally, (c) if leader-member exchange (LMX) is associated with this transition. We use LTA to examine these steps in a German sample (*N* = 180).

## Introduction

The field of Organizational Psychology (OP) is undergoing tremendous changes. The number of scholars continues to grow (e.g., SIOP Membership Committee, 2020), and it is one of the fastest-growing fields in psychology regarding job opportunities (The Bureau of Labor Statistics, 2022). Similarly, these changes were accompanied by various data collection and analysis methodologies. For example, Cortina et al. (2017) refer to the timeframe between 1990 and 2014 as “from one to many” (p. 280) in their review of OP research methods, indicating the increased methodological alternatives for data analyses with a noteworthy increase in longitudinal studies (Rigby and Traylor, 2020).

Longitudinal designs help OP researchers to understand and predict behaviors over time rather than a specified time point (Ployhart et al., 2002). Furthermore, longitudinal data analyses can address the problem of common method variance associated with cross-sectional survey data (Podsakoff et al., 2003; Cooper et al., 2020) and establish stronger causal claims through temporal precedence (Zapf et al., 1996; Spector, 2019; Vander Weele et al., 2020). However, despite advantages, the proportion of studies employing longitudinal designs over cross-sectional surveys remains scarce (Schein, 2015), reasons for which vary across domains. For instance, Ployhart et al. (2002) noted that conceptual and methodological issues remain a key challenge for the leadership domain, explaining the underrepresented usage of longitudinal designs.

A significant development in the methodological aspect of longitudinal designs is the variety of methods OP researchers can use to analyze longitudinal data, for example, random coefficient modeling (RCM; Ployhart et al., 2002), discontinuous growth models (DGM; Bliese et al., 2017), and growth mixture models (GMM; Muthén and Muthén, 2000). In this study, we present a non-technical tutorial for analyzing longitudinal data by focusing on latent transition analysis (LTA) and follow the lead of prominent researchers encouraging non-technical demonstrations of new methodologies in psychological science for broader application (e.g., Aiman-Smith et al., 2002; Ployhart et al., 2002; Marsh and Hau, 2007; Morin et al., 2018; Aguinis et al., 2020).

LTA is a person-centered approach to grouping individuals into different profiles based on several variables. Individuals with the same profile are similar regarding the “score” on the variables and different from those in other categories (Muthén and Muthén, 2000). The main aim of LTA is to model the probability of individuals’ transitioning from a profile at one point in time to another profile at another point. For example, across five lessons, Schlatter et al. (2021) demonstrated transition probabilities of fifth-graders from a low-achiever group to the high-achiever one. Although LTA has been increasing as a popular statistical technique among researchers for the analyses of longitudinal data, Woo et al. (2018) identified only four articles that utilized LTA in OP research from 1998 to 2016. Since then, numerous articles employing LTA have been published, yet we believe that LTA has still not received the attention it deserves from OP researchers because of the common technical language used in LTA tutorials.

In this paper, we explain LTA in a non-technical way with an empirical example to encourage OP researchers to apply it in their research. In the following, we first introduce LTA, differentiate it from other person-centered approaches, point to questions researchers could answer using LTA, and review the OP literature to summarize the LTA literature. With the aims mentioned, we contribute to the OP field in several ways. First and foremost, we encourage OP researchers to adopt and introduce LTA through using a simplified, non-technical language and presenting an applied example. Second, we sample questions for the researchers new to LTA to be answered using it. Finally, we give an up-to-date account of the OP literature that employed LTA as a data analytical approach. We share the applied code and data for this LTA introduction to foster open and transparent research.

### LTA is a person-centered approach

Researchers in OP have widely used variable-centered approaches such as regression analysis, factor analysis, and structural equation modeling for data analyses. Such approaches aim to examine the relationship between two variables and to predict outcome variables from specific predictors. Such analyses are commonly referred to as variable-centered approaches presuming that the individuals composing a specific sample share the same population, and thus, a single parameter for the sample is estimated. On the other hand, person-centered approaches, such as LTA, assume that there might be more than one population (i.e., multiple subpopulations) in the sample, and these subpopulations might have different sets of parameters (Morin et al., 2018). This difference also provides insights into the main aim of person-centered approaches, categorizing individuals from a sample into groups with different profiles based on a set of variables. Individuals with the same profile are said to be similar in terms of the variables they are measured on and different from those belong to the other profiles (Muthén and Muthén, 2000).

LTA is one of the many approaches clustered together under the umbrella term person-centered approaches. LTA describes the changes in latent profiles estimated from the probabilities of a set of variables to vary across groups of individuals over different time points. Specifically, LTA uses longitudinal data and examines the transition probabilities of individuals from a latent profile at a particular time point to other latent profiles at the next time point (Muthén and Muthén, 2000). To better understand LTA, we refer to the differences between person-centered approaches in the following.

### A brief overview of some person-centered approaches

#### Latent profile (class) analysis

Latent profile analysis (LPA) is a person-centered approach that aims to establish profiles or classes of individuals with different configurations on personal and/or environmental variables (Spurk et al., 2020; Bakaç et al., 2021). By relying on a set of variables measured (typically) on a cross-sectional basis, LPA produces latent profiles that reveal associations among a set of variables (Muthén and Muthén, 2000; Woo et al., 2018). The LPA adds more classes stepwise until a well-fitted model is found (Muthén and Muthén, 2000). Relevant is the model, which assigns each individual an estimated probability of belonging to different profiles. Researchers have analogously used the terms LPA and latent class analysis (LCA). The difference between the two is that the former uses continuous indicators or variables, but the latter utilizes dichotomous or polytomous variables (Woo et al., 2018). In this paper, we also use them interchangeably and refer to them as LPA.

#### Latent transition analysis

Like LPA, LTA computes latent profiles based on a set of variables and assigns individuals a probability of belonging to each profile. However, LTA is a model used for longitudinal data and estimates the likelihood of individuals transitioning from a specific profile at one time to other profiles at the next time point (Wang and Hanges, 2011). Furthermore, LPA can model the covariates of profile transition over time to investigate variables that might explain the transition (e.g., Huyghebaert-Zouaghi et al., 2022). For example, Kam et al. (2016) examined how employees’ commitment profiles changed over 8 months and added perceived management trustworthiness to predict employees’ profile transition.

#### Growth mixture modeling

Growth mixture modeling (GMM) is another person-centered approach for analyzing longitudinal data (Muthén and Muthén, 2000). It takes its main functionalities from conventional growth models, which analyze longitudinal data by relating an outcome variable to a time or time-related variable like age. Individuals’ growth trajectories, modeled by letting the coefficients for each individual, vary. While traditional growth models establish a mean growth estimate for the sample (e.g., Muthén and Muthén, 2000; Jung and Wickrama, 2008), the assumption that individuals composing the sample come from the same population and have a mean growth estimate is relaxed in GMM. That is, GMM does not impose a mean growth estimate on the complete sample but lets the sample have subgroups with their growth estimates within the sample. Thus, growth trajectories can change across some latent profiles in that each latent profile has its estimates of variances and covariates its influences (Jung and Wickrama, 2008). An example study for GMM comes from Phelps et al. (2018), who modeled veterans with post-traumatic stress disorder regarding their treatment response trajectories and used depression to predict the differences in these trajectories.

#### Latent class growth analysis

Latent class growth analysis (LCGA) is a specialized version of GMM. The main difference between LCGA and GMM is that the variance and covariance estimates of growth factors are constrained to zero within each profile in LCGM (Jung and Wickrama, 2008). With this constraint, all individuals within a profile have the same growth trajectories. Collecting data from employees with a diary study, Mühlenmeier et al. (2021) investigated the time pressure trajectories and changes in these trajectories from the end of a working week until the start of the next working week. They also examined how these trajectories differ in terms of employee well-being.

For more details on the models specified above, we encourage the readers to see Jung and Wickrama (2008), Muthén and Muthén (2000), Morin et al. (2011), and Sorgente et al. (2019).

### What kinds of questions does LTA answer?

LTA can be used to answer questions regarding changes in individuals’ profile transition over time. Specifically, researchers might investigate if individuals belonging to a specific profile at one time point transit to another profile at time two and its possible/likely predictors and consequences. Compared to LPA, the focus is not on the profile membership but rather on the transition in profile membership. For example, personality researchers might be interested in individuals’ Big-Five personality profile stability across time, assuming no transition among profiles. However, individuals can also transit from one specific personality profile to another due to critical life events such as unemployment (Boyce et al., 2015). It is also possible to experimentally intervene in the transition. To illustrate, researchers could experimentally manipulate the potential predictors of change and observe the effects of this manipulation on the transition of individuals from “undesirable” to “desirable” profiles. For instance, Liao et al. (2018) conducted an experiment to study the effect of the availability of a business model on individuals’ electric car adoption. By conducting a latent transition analysis of car preferences, they investigated the transition in car preference membership after a particular business model was available.

## Conducting LTA: A five-step approach

The classical way of analyzing data with person-centered approaches is to combine all of the five-steps we describe below (i.e., latent transition analysis, predicting the outcome variables with the transition probabilities, etc.) in one model. However, researchers have criticized this approach. For example, Vermunt (2010) mentioned some of the disadvantages of the approach, such as the difficulty of deciding the number of profiles with or without the included covariates. Vermunt (2010) and Asparouhov and Muthén (2014) suggested a three-step approach in such analyses, including LTA, each of which to be estimated separately. Here, we refer to the three-step approach, follow Ryoo et al. (2018) guidelines, and add two additional steps to ease the understanding.

### Step 1: Estimate latent profiles for each measurement point separately

Before commencing with the LPA/LTA analyses, we strongly encourage researchers to diagnose their data for possible anomalies and check the descriptive statistics for each measurement point in the data separately. After this initial diagnosis, researchers first identify the number of profiles to retain each time point separately using LPA (Ryoo et al., 2018). The decision about the number of profiles is generally based on statistical fit parameters such as Bayesian information criterion (BIC), Akaike Information Criterion (AIC), sample-adjusted BIC (SABIC), Bootstrap Likelihood Ratio Test (BLRT), and Entropy (Nylund et al., 2007). Among these parameters, BIC is the most robust model fit indicator. For instance, Spurk et al. (2020) found that most researchers use BIC as a key indicator. Ram and Grimm (2009) recommended lower values on the AIC, BIC, and ABIC, along with significant BLRT and higher Entropy values to indicate better-fitting solutions.

### Step 2: Testing longitudinal measurement invariance using LTA

Ryoo et al. (2018) suggest that testing the longitudinal measurement invariance *via* LTA is an important step. Because the next step depends on whether the measurement across time points is invariant, it is desirable to demonstrate that the observed variables estimate the latent profile characteristics over time. Ryoo et al. (2018) used the likelihood ratio test difference to measure measurement invariance, which we also use in the applied example.

### Step 3: Defining qualitatively distinct profiles

Although this step is generally combined with other steps (Ryoo et al., 2018), we think it is important to distinguish it from the other steps because it would make it easier for researchers to follow. In this step, a researcher should label the latent profiles based on variable probabilities to document the existence of qualitatively distinct profiles. These are also known as the shape differences between profiles (e.g., Spurk et al., 2020), where shape primarily concerns the mean differences between profiles (i.e., high/low levels below or above the mean). Noteworthy, labeling the profiles can be done based on the level of each variable within the profile (e.g., low vs. high) to describe the respective profile. However, in case of many profiles, one can assign numbers (e.g., Profile 1, Profile 2) rather than labels (Spurk et al., 2020).

### Step 4: Estimate status prevalence and transition probabilities

One of the most critical steps in conducting LTA is defining latent status prevalence and estimating the transition probabilities. Latent status prevalence gives the percentage of individuals across profiles. Furthermore, transition probabilities describe the probability of transitioning from a specific profile at one time to all the other profiles at the next time point. For example, an LTA across two time points may result in five profiles in Time 1 and the latent status prevalence in one of the profiles (e.g., Profile 5) may be 13% for Time 1 and 12% for Time 2. Furthermore, the transition probability of individuals from Profile 5 at Time 1 to Profile 5 at Time 2 may be 0.97. Based on these results, one might conclude that the percentage of individuals in Profile 5 did not change much between times, showing that individuals did not transition to other profiles between times. Thus, individuals in Profile 5 remained in the same profile across both time points (Nylund-Gibson et al., 2014).

### Step 5: Adding covariates and/or moderators

Conducting person-centered approaches like LTA, researchers are often interested in adding outcome and predictor covariates as well as moderators to the model. When adding a covariate as a predictor, researchers could test whether the covariable is associated with a specific profile or the transition is from one profile at Time 1 to another profile at Time 2. In the former, researchers test if the added variable predicts the profile membership. In the latter, researchers examine if the added variable is significantly associated with the inter-profile transition of individuals. When adding a covariate as an outcome variable it is possible to investigate if the profile transition is significantly associated with the outcome variable. For instance, researchers can examine differences in the outcome variable between people who transitioned from one profile to another and people who stayed in the same profiles. Furthermore, it is possible to test if the profile transition probabilities depend on a moderator variable. For example, Vaziri et al. (2020) established work-family profiles and investigated leader compassion as a predictor and turnover intentions as an outcome variable of profile transition.

## An applied example

Below we present an applied example of how to conduct LTA. In this example, we use Psychological Capital (PsyCap) to investigate sub-group profiles of employees and their stability (i.e., change or not) across two waves. Furthermore, we use Leader-Member-Exchange (LMX) as a possible variable associated with the stability of the PsyCap profiles across the two waves. Finally, we provide an overview of PsyCap and LMX and their implications and derive the research questions, which can be used as an example for the OP researchers.

### PsyCap profiles and LMX as a predictor of their transition

PsyCap is a positive psychological resource comprised of four mechanisms or dimensions: self-efficacy, hope, optimism, and resilience (Luthans et al., 2007). Self-efficacy concerns individuals’ beliefs about their skills and abilities to successfully mobilize resources to execute tasks (Bandura et al., 1999). Hope is a motivational state guided primarily by goal-directed energies based on the predictions of some positive outcomes (Luthans et al., 2006). Optimism is a positive psychological resource defined as an attributive style that facilitates interpreting adverse events as temporary and situation-specific (Avey et al., 2011). Finally, resilience is a robust positive psychological state linked to important job-related outcomes and defined as a “psychological capacity to recover from adversity, uncertainty, conflict, failure, or even positive change, progress and increased responsibility” (Luthans et al., 2006, p. 702). The authors of PsyCap (Youssef-Morgan and Luthans, 2015) referred to these four mechanisms as the “HERO within” (Hope, Efficacy, Resilience, and Optimism), claiming they act as a single body in facilitating the various positive and negative experiences of individuals.

PsyCap as a personal construct has gained significant attention from scholars in various fields, including organizational and management (Liu, 2021; Dirzyte and Patapas, 2022). Research showed that PsyCap could be relevant for many job-related outcomes, such as job satisfaction (Zhang et al., 2021), innovation (Dimas et al., 2022), and problem-solving (Ho and Chan, 2022), as well as health performance, such as reducing emotional exhaustion (Jung and Yoon, 2022) and increasing overall mental health (Cao et al., 2022). Current findings have shown that PsyCap helps individuals deal with challenging environments, such as the COVID-19 crisis (Alat et al., 2021; Zyberaj et al., 2022). For instance, Zyberaj et al. (2022) found positive associations between PsyCap and career satisfaction and coping during the COVID-19 pandemic. Compared to previous research which investigated the role of personality characteristics (e.g., the Big-Five) separately (Iliescu et al., 2017), PsyCap mechanisms act as a single body (Luthans and Youssef-Morgan, 2017). Thus, it is crucial to study PsyCap with a person-centered approach to consider the combination of the four PsyCap mechanisms within an individual.

We also investigate LMX because it represents another essential concept in organizational psychology (Graen and Uhl-Bien, 1995; Martin et al., 2016). According to the dyadic theory of LMX (Graen and Uhl-Bien, 1995; Bauer and Erdogan, 2015), leaders behave toward their employees differently based on relationship quality. Thus, unlike other leadership theories, which emphasize the individual leader (i.e., leader-centric focused), LMX is unique in its focus on the dyad (i.e., supervisor-subordinate). In meta-analytic research, Martin et al. (2016) have shown that a high-quality LMX relationship is essential for the attitudes and performance of employees. For instance, LMX has been found to be related to employees’ proactive behavior (Lai et al., 2019), engagement and job satisfaction (Volmer et al., 2011; González-Navarro et al., 2019), as well as their resilience (Kakkar, 2019). Moreover, González-Navarro et al. (2019) found a positive association between LMX and job engagement. Research has also shown that PsyCap can be significantly enhanced through proximity, trust, and support that leaders can provide their employees with (Law et al., 2010; Kakkar, 2019) and that LMX-quality can predict PsyCap profiles (Luthans et al., 2004).

Based on the research findings above and the exploratory approach of our research, we will answer the following research questions:

#### Research question 1

Does latent transition analysis reveal quantitatively and qualitatively distinct PsyCap profiles?

#### Research question 2

Do individuals stay in the same PsyCap profile across time or transit from one profile to another?

#### Research question 3

Is LMX significantly associated with the PsyCap profile transition?

### The present study

With this study, we aim to further enhance our understanding of the LTA research methodology for OP through an applied example. For this, we use PsyCap to create employee profiles and employ LMX as a possible predictor of the PsyCap profile transitions.

Guided by an exploratory approach, we aim to investigate the membership of employees in the four mechanisms of the PsyCap. However, because PsyCap mechanisms are reported to be relatively stable across time, we expect PsyCap profile transitions to remain stable across the two measurement points. One study conducted profile analysis using PsyCap (Bouckenooghe et al., 2019); however, unlike our approach, Bouckenooghe et al. (2019) employed a latent profile analysis (LPA), which differs concerning the LTA in its cross-sectional approach. Therefore, with our LTA approach, we add to PsyCap profiles by reducing common method bias and providing insights into the PsyCap profiles’ robustness across different time points.

### Materials and methods

#### Participants

We conducted our study during the summer and autumn of 2021. We recruited our sample through the SoSci Panel.^1^ The panel includes German-speaking samples, majority from Germany and some from Austria and Switzerland. Not being interested in country-specific data, we did not ask participants about their exact country. Initially, 310 and 303 surveys were filled fully at Time 1 (T1) and Time 2 (T2), with a gap of 6 weeks between the time points. However, after excluding the individuals who missed measurement points (listwise deletion of cases), our final sample included 185 participants (81 females). The age ranged from 19 to 67 (*M_age_* = 43.61, *SD_age_* = 12). The majority of the participants held a bachelor’s or equivalent degree (40.54%), lived with a partner (43.24%), had no children (61.08%), worked full-time (67.02%), and worked 1 day per week from home (37.30%). On average, participants have worked in their companies for 12.84 years (*SD* = 10.49).

#### Measures

##### Psychological capital

We measured PsyCap using the 12-item short version of the Psychological Capital Questionnaire (PCQ; Luthans et al., 2007). The German version of the questionnaire was provided by Mind Garden.^2^ Recent research has shown this measure to be solid and robust (Kauffeld and Spurk, 2022). The scale assesses all four mechanisms of PsyCap. There are three items used for measuring self-efficacy. A sample item is “I feel confident in representing my work area in meetings with management”). In addition, four items are employed for hope. A sample item is “If I should find myself in jam at work, I could think of many ways to get out of it.” Resilience is measured with three items. One sample item is “I usually take stressful things at work in stride.” Finally, optimism is measured with two items. Sample item is “I always look on the bright side of things regarding my job.” PsyCap uses a 6-point rating scale with 1 (*strongly disagree*) to 6 (*strongly agree*). The Cronbach’s alphas of the PsyCap dimensions (i.e., self-efficacy, hope, resilience, and optimism) were 0.70, 0.77, 0.67, and 0.74 for T1 and 0.79, 0.79, 0.66, and 0.79 for T2, respectively.

##### Leader-member exchange

We used the German version (Schyns, 2002) of the original LMX-7 scale developed by Graen and Uhl-Bien (1995). This measure contains seven items, and participants rate each item on a 5-point Likert-type scale with the anchors adapted to the respective item between 1 (*never*) and 5 (*always*). A sample item is “I have enough confidence in my supervisor to defend his/her decisions.” Cronbach’s alpha was 0.94 (T2).

### Analyses

We conducted LTA analyses using Mplus 8.4 (Muthén and Muthén, 2017), following the steps specified above. The data and codes for analyzing the data are publicly accessible on Open Science Framework (OSF).^3^ For each step of the analysis, we provide a Mplus script with comments.

First of all, we conducted two LPAs: one for each time point, to decide on the number of profiles to retain at each time point. After that, we conducted two LTAs, one with measurement invariance constraints and one without any constraints. We compared the two models using the likelihood ratio test to test if measurement is invariant across time points. If the measurement is not invariant, models without measurement invariance constraints are run for further analyses. However, we provide Mplus code with and without measurement invariance constraints for researchers to analyze their data.

Furthermore, we computed an LTA to document the transition probabilities from profiles estimated at T1 to profiles estimated at T2. Finally, we added a moderator, namely LMX measured at T2, to investigate if the transition probabilities are conditional upon LMX. Specifically, we calculated conditional transition probabilities for high and low levels of LMX (± 1SD; Muthén and Asparouhov, 2011). To determine if the conditional transition probabilities at different levels of LMX differed from each other, we used the delta method (Raykov and Marcoulides, 2004).

### Results

#### Descriptive statistics

Table 1 displays descriptive statistics and the zero-order correlations among study variables. Correlations between the PsyCap dimensions and LMX were positive and ranged from 0.11 (*p* > 0.05) to 0.32 (*p* < 0.01). Moreover, there were medium-high correlations among dimensions of PsyCap, ranging from 0.33 (*p* < 0.01) to 0.60 (*p* < 0.01). The correlations of dimensions with themselves measured at different time points were also high and significant.

**Table 1:** Means, standard deviations, and correlations.

Variable	*M*	*SD*	1	2	3	4	5	6	7	8
1. LMX	3.40	1.02								
2. Efficacy T1	5.04	0.87	0.11							
3. Hope T1	4.71	0.86	0.29**	0.47**						
4. Resilience T1	4.71	0.90	0.14	0.55**	0.71**					
5. Optimism T1	4.03	1.19	0.26**	0.34**	0.54**	0.50**				
6. Efficacy T2	5.02	0.87	0.13	0.69**	0.44**	0.50**	0.31**			
7. Hope T2	4.78	0.81	0.22**	0.40**	0.69**	0.53**	0.46**	0.60**		
8. Resilience T2	4.74	0.86	0.15*	0.44**	0.47**	0.66**	0.35**	0.55**	0.60**	
9. Optimism T2	4.05	1.16	0.32**	0.33**	0.42**	0.42**	0.74**	0.38**	0.58**	0.53**

*N* = 185. LMX, leader-member exchange.

*p < 0.05; **p < 0.01.

#### Latent transition analysis

##### Step 1: Estimate latent profiles for each measurement point separately

**Figure 1:**
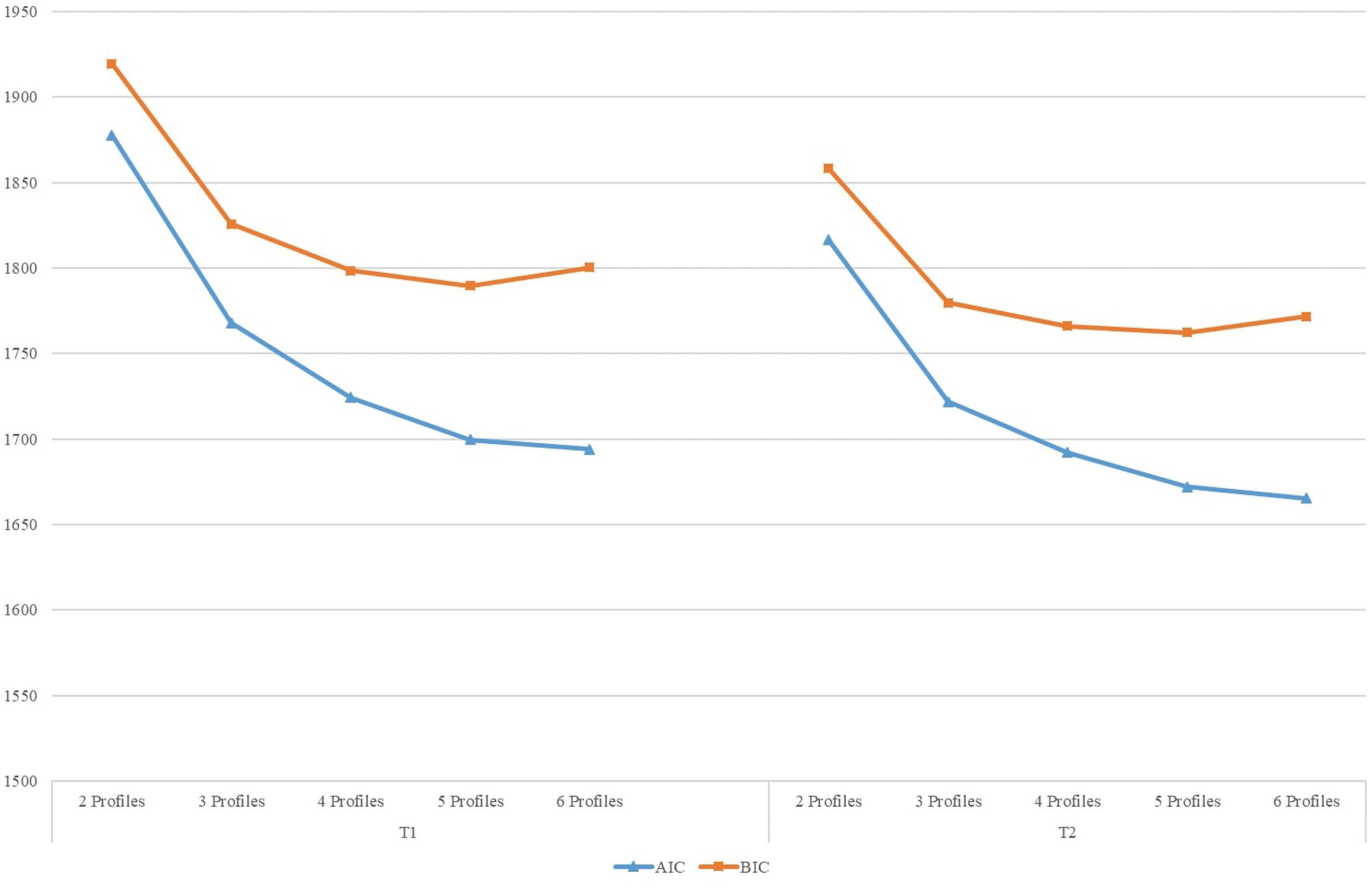
Elbow plot for BIC and AIC in determining profile solution. BIC, Bayesian information criterion; AIC, Akaike information criteria.

**Figure 2:**
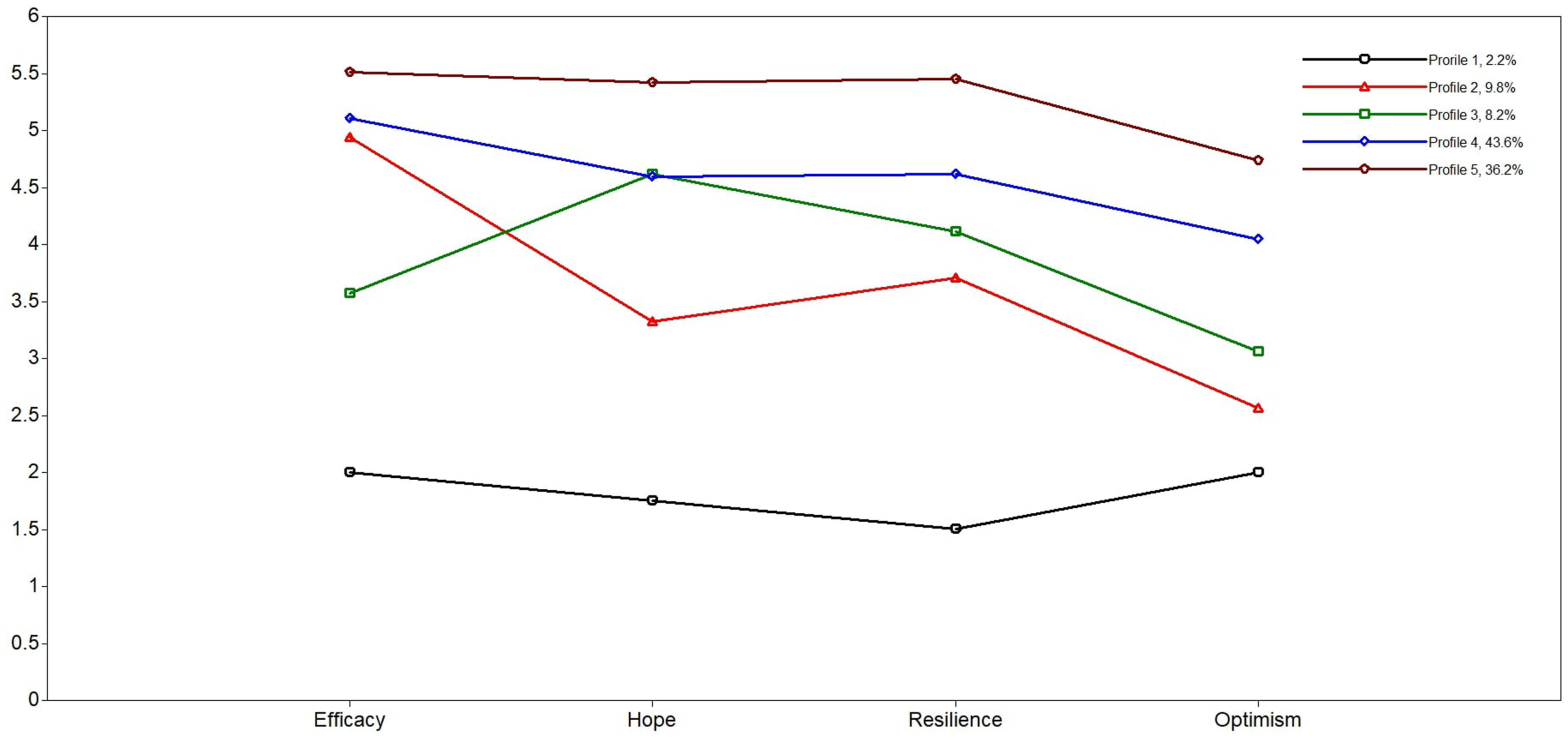
Latent profiles of PsyCap dimensions at T1 and the profile percentages. Profile 1: *Low PsyCap*; Profile 2: *Dominant Efficacy PsyCap*; Profile 3: *Dominant Hope PsyCap*; Profile 4: *Moderate High PsyCap*; Profile 5: *High PsyCap*.

In the first step, we entered the mean of the four facets of PsyCap to estimate profiles for T1 and T2. Table 2 shows the results from LPA. Based on the fit indices with the lowest BIC values, a significant LMR (*p*) value, and high Entropy (higher values indicate higher confidence in the model), five profiles were retained at T1. However, relatedly, T2 did not provide strongly differentiated fit indices. Here, we also decided to retain five profiles because BIC is the lowest, especially due to the small sample size (e.g., Magidson and Vermunt, 2004; Nylund et al., 2007). Additionally, the Elbow plot (Figure 1) demonstrated that the slopes receded around the five-profile solution, further supporting our decision. To depict the retained profiles, we plotted them using the ones from T1, which are similar to profiles in T2 (see Figure 2). As Figure 2 shows, Profile 5 scored relatively high in all PsyCap mechanisms, Profile 1 scored relatively low, Profile 2 scored medium-high and low, Profile 4 scored medium-high, while Profile 3 tended to remain around the medium level. See step three below for the names we assigned to these profiles.

**Table 2:** Fit indices and number of profiles for T1 and T2.

# of profiles	LL	FP	AIC	BIC	SABIC	LMR(*p*)	Entropy
T1							
2	−1009.00	13	1877.65	1919.52	1878.34	0.30	0.91
3	−925.83	18	1767.88	1825.85	1768.84	<0.05	0.82
4	−865.94	23	1724.41	1798.48	1725.63	<0.05	0.85
**5**	**−839.20**	**28**	**1699.54**	**1789.71**	**1701.02**	**<0.05**	**0.85**
6	−821.77	33	1693.96	1800.23	1695.71	0.51	0.84
T2							
2	−981.37	13	1816.47	1858.33	1817.16	0.43	0.88
3	−895.23	18	1721.85	1779.82	1722.81	<0.001	0.85
4	−842.93	23	1692.06	1766.12	1693.28	0.64	0.77
**5**	**−823.03**	**28**	**1672.03**	**1762.20**	**1673.52**	**0.12**	**0.77**
6	−808.02	33	1665.49	1771.76	1667.24	0.19	0.79

*N* = 185. Bold font indicates selected models. LL, log likelihood; FP, free parameters; AIC, Akaike information criteria; BIC, Bayesian information criteria; SABIC, sample-size adjusted BIC; LMR(*p*), value of *p* for the Lo et al. (2001) test.

##### Step 2: Testing longitudinal measurement invariance using LTA

We computed measurement invariance by computing two LTAs, one with invariance constrained and one without the invariance constrained. Specifically, we computed the measurement invariant model by constraining the variable-response probabilities to be equal at both times and measurement non-invariant model by freely estimating variable-response probabilities at each time. We compared the likelihood ratios of the two models to investigate if the added constraints affect the model fit. The likelihood ratio difference test demonstrated that the models differed significantly (*G^2^_Δ_* = 49.74, *df_Δ_* = 20, *p* < 0.05). Thus, adding measurement invariance constraint changed the model significantly from the model without the constraint, indicating a measurement non-invariance across time points. As we indicated earlier, we conducted our analyses with measurement invariance constraints for practical reasons and provide Mplus code for the implementation of non-measurement invariance constraints as well.

##### Step 3: Defining qualitatively distinct profiles

To name profiles, we compared them to each other in terms of PsyCap dimensions using ANOVAs with *post-hoc* t-tests with Bonferroni corrections. The means and standard deviations of profiles across the PsyCap dimensions are presented in Table 3. T-test results showed that Profile 5 was significantly higher than other profiles across all PsyCap dimensions. Profile 1 was significantly lower than the other profiles across all PsyCap dimensions except for optimism. However, Profile 1 did not differ from Profile 2 and 3. Furthermore, Profile 4 was significantly higher than Profile 2 in terms of hope, resilience, and optimism but not efficacy and significantly higher than Profile 3 in terms of efficacy, resilience, and optimism but not hope. Profile 3 was significantly lower than Profile 2 on efficacy, higher on hope, and not different on resilience and optimism. Based on these and raw mean values of PsyCap facets for each profile, we named the profiles *Low PsyCap*, *Dominant Efficacy PsyCap*, *Dominant Hope PsyCap*, *Moderate High PsyCap*, and *High PsyCap* for Profile 1, 2, 3, 4, and 5, respectively.

**Table 3:** Means and standard deviation of PsyCap dimensions across profiles at Time 1.

Profiles	Efficacy		Hope		Resilience		Optimism	
	*M*	*SD*	*M*	*SD*	*M*	*SD*	*M*	*SD*
Low PsyCap	2.00	0.27	1.75	0.46	1.50	1.04	2.00	1.41
Dominant efficacy PsyCap	4.93	0.64	3.34	0.47	3.70	0.48	2.53	1.05
Dominant hope PsyCap	3.50	0.59	4.66	0.47	4.05	0.57	2.96	0.97
Moderate high PsyCap	5.12	0.50	4.56	0.40	4.60	0.53	4.06	0.86
High PsyCap	5.48	0.60	5.46	0.32	5.46	0.40	4.75	0.88

*N = 185*.

##### Step 4: Estimate status prevalence and transition probabilities

Table 4 shows the transition probabilities of profiles from T1 to T2. Transitions from profiles estimated at T1 were mostly estimated at T2 as well. However, there are also interesting transitions among profiles. For example, the transition probability from Profile 1 estimated at T1 to Profile 4 estimated at T2 is 0.50, meaning that the probability that individuals at Profile 1 at T1 transit to Profile 4 at T2 is 0.50. Further details can be found in Table 4.

**Table 4:** Profile transition probabilities.

	Profiles at T2				
Profiles at T1	Profile 1	Profile 2	Profile 3	Profile 4	Profile 5
1 Low PsyCap	0.50	0.00	0.00	0.50	0.00
2 Dominant efficacy PsyCap	0.00	1.00	0.00	0.00	0.00
3 Dominant hope PsyCap	0.00	0.068	0.932	0.00	0.00
4 Moderate high PsyCap	0.053	0.047	0.00	0.705	0.195
5 High PsyCap	0.00	0.052	0.00	0.104	0.843

**Figure 3:**
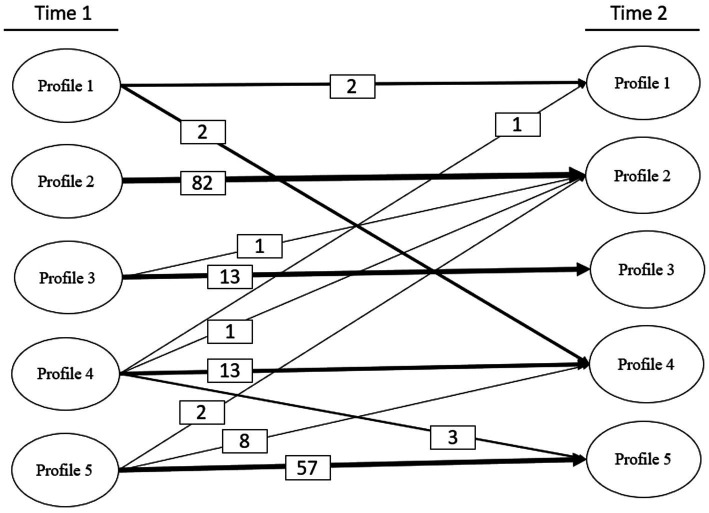
Transition paths and prevalence. Thicker lines indicate higher transition prevalence. Values (i.e., numbers in the small boxes) indicate the number of people transitioning between profiles from T1 to T2.

In Figure 3, we additionally demonstrate the transition paths and prevalence. Thicker lines indicate higher transition probabilities and prevalence. As shown, there was no transition from Profile 2 estimated at T1 to other profiles estimated at T2. In other words, people in Profile 2 remain in Profile 2 across T1 and T2. However, eight people transitioned from Profile 5 estimated at T1 to Profile 4 estimated at T2. For further details, see Figure 3.

##### Step 5: Adding a moderator

To investigate if transition probabilities depended on different levels of LMX (i.e., high vs. low), we conducted a conditional LTA. Specifically, we set LMX to ± one standard deviation to compute high and low values of LMX and used the delta method (Raykov and Marcoulides, 2004; Vaziri et al., 2020) to examine if transition probabilities differed from each other at different levels of LMX. Delta method is an “… analytic approach for approximate standard error and confidence interval construction” (Raykov and Marcoulides, 2004, p. 634). The results are presented in Table 5. For each path, we provide the transition probability for high and low values of LMX and indicate if there was a difference between the two levels in transition probability. The results showed that the transition probability of Profile 5 (T1) to itself measured at T2 was significant both for low values of LMX (0.54, *p* < 0.05) and for high values of LMX (0.89, *p* < 0.01), and this transition probability differed between the two significantly. In other words, in Profile 5, there was a higher transition probability for individuals who reported a high LMX. For further details, see Table 5.

**Table 5:** Conditional transition probabilities across lower and higher levels of LMX.

Transition path	LMX		Difference
	Low	High	
Low PsyCap (no transition)	0.79**	0.93**	n.s.
Low PsyCap ➛ Dominant efficacy PsyCap	0.00	0.00	n.s.
Low PsyCap ➛ Dominant hope PsyCap	0.00	0.00	n.s.
Low PsyCap ➛ Moderate high PsyCap	0.09	0.01	n.s.
Low PsyCap ➛ High PsyCap	0.11	0.05	n.s.
Dominant efficacy PsyCap ➛ Low PsyCap	0.00	1.00**	<0.001
Dominant efficacy PsyCap (no transition)	1.00**	0.00	<0.001
Dominant efficacy PsyCap ➛ Dominant hope PsyCap	0.00	0.00	n.s.
Dominant efficacy PsyCap ➛ Moderate high PsyCap	0.00	0.00	n.s.
Dominant efficacy PsyCap ➛ High PsyCap	0.00	0.00	n.s.
Dominant hope PsyCap ➛ Low PsyCap	0.00	1.00**	<0.001
Dominant hope PsyCap ➛ Dominant efficacy PsyCap	0.00	0.00	n.s.
Dominant hope PsyCap (no transition)	0.93**	0.00	<0.001
Dominant hope PsyCap ➛ Moderate high PsyCap	0.07	0.00	n.s.
Dominant hope PsyCap ➛ High PsyCap	0.00	0.00	n.s.
Moderate high PsyCap ➛ Low PsyCap	0.00*	0.00*	<0.05
Moderate high PsyCap ➛ Dominant efficacy PsyCap	0.00	0.00	n.s.
Moderate high PsyCap ➛ Dominant hope PsyCap	0.00	0.00	n.s.
Moderate high PsyCap (no transition)	1.00*	1.00**	n.s.
Moderate high PsyCap ➛ High PsyCap	0.00	0.00	n.s.
High PsyCap ➛ Low PsyCap	0.39*	0.09	<0.05
High PsyCap ➛ Dominant efficacy PsyCap	0.07	0.02	n.s.
High PsyCap ➛ Dominant hope PsyCap	0.00	0.00	n.s.
High PsyCap ➛ Moderate high PsyCap	0.00	0.00	n.s.
High PsyCap (no transition)	0.54*	0.89**	<0.05

LMX, leader-member exchange.

*p < 0.05; **p < 0.001.

In this paper, we did not consider any distal outcomes or predictors of latent profiles. However, we provide MPlus code on the respective OSF directory.

### Discussion

We found five different profiles for PsyCap mechanisms at Time 1 and the same five profiles at Time 2. These different profiles pointed out that the mechanisms explaining the impact of PsyCap on several beneficial outcomes (e.g., for better mental health, Cao et al., 2022) might not be the same for all individuals. Especially, Profile 2 (Dominant Efficacy PsyCap) and Profile 3 (Dominant Hope PsyCap) demonstrated that different patterns of PsyCap exist. For instance, two employees might have the same medium level of PsyCap but differ in the combination of the four mechanisms. We found PsyCap profiles with high hope or efficacy, yet no profiles with either high resilience or optimism. Thus, one may speculate that for an average level of PsyCap, high levels of hope or efficacy are sufficient, while at the same time, a certain amount of optimism and resilience is necessary.

Our results indicated that most individuals have a stable profile (i.e., no transition) across 6 weeks, which concurs with PsyCap theory (Luthans and Youssef-Morgan, 2017). However, essential transitions emerged from Profile 5 (High PsyCap) to Profile 4 (Moderate High PsyCap), which indicates a small stable decrease in PsyCap. Our results add to the PsyCap theory noting the significance of the PsyCap mechanisms in acting as a single body and a whole-person perspective on personality. Because PsyCap mechanisms have emerged from the positivity literature, their stability across time can be explained by one core assumption of the PsyCap theory, namely: the reciprocity of hope, efficacy, optimism, and resilience (Luthans et al., 2007). According to the PsyCap theory, “reciprocity indicates that each variable causally influences and is causally influenced by the others” (Youssef-Morgan and Luthans, 2013; p. 151). Thus, PsyCap profiles might benefit from strong reciprocal interactions between the mechanisms and these strong connections between the mechanisms might lead to the stable profiles we identified in LTA.

Moreover, our results align with previous research showing a similar pattern of PsyCap profiles. For instance, a study by Bouckenooghe et al. (2019) found a similar pattern in profiles with one additional profile.

We also demonstrated that LMX was associated with the transition probabilities for specific profiles, with the most interesting results for the no transition probability for Profile 5. Because Profile 5 refers to very high levels of PsyCap, individuals with that profile should not transit, supported by high LMX. These results align with previous research that showed positive associations of LMX with various job-and personality-related variables, such as proactive behaviors (Lai et al., 2019) and resilience, a core mechanism of PsyCap (Kakkar, 2019). Similarly, research has also noted that LMX features, such as trust and proximity, can also enhance one’s PsyCap (Law et al., 2010; Kakkar, 2019). Regarding Profile 5, it is important to note that the role of LMX is not always robust, especially across contexts. For instance, a study by Zyberaj et al. (2022) found no moderating role of LMX between PsyCap mechanisms and employees’ career adaptability during crises. As the authors note, “employees high in PsyCap have strong psychological resources that can facilitate their functioning in challenging environments (e.g., adaptability), even when there is a lack of social support” (p. 12). Similar to the assumption of reciprocity noted by the PsyCap theory (Youssef-Morgan and Luthans, 2013), the robustness of Profile 5 might be explained by the assumption that PsyCap mechanisms facilitate one’s functioning through their acting as a single body and a whole-personality approach.

It is noteworthy that the results from our illustrative example should be treated with caution because of concerns regarding the LMX theory (e.g., Antonakis et al., 2014; Antonakis, 2017). Antonakis (2017) voiced that LMX is riddled with many issues and shares a lot of commonalities in terms of common causes with the constructs it predicts. That is, LMX is an endogenous construct, sharing common causes with the psychological constructs used as outcome variables.

## General discussion

Our applied example showed that LTA helps researchers to answer three research questions: (1) Do different profiles of variable combinations exist (e.g., PsyCap mechanisms), (2) do these profiles change from one measurement point to another (e.g., from T1 to T2), and (3) which variables (e.g., LMX) might explain this transition. With these three general questions in mind, one may think of several fields of OP where the use of LTA is beneficial.

### Usage and significance of the LTA for OP

The first step of the LTA, identifying the profiles, helps shift the view from a variable-centered to a person-centered approach. Instead of investigating how variables are related, the profiles indicate how a set of variables “describes” a person. Thus, researchers could study how different combinations of related or similar constructs manifest within one person. The results may help get a deeper understanding of how a combination of the constructs’ facets drives an individual’s behavior. For instance, instead of comparing job satisfaction at a global or facet-oriented level, OP researchers could examine whether different satisfaction profiles exist (e.g., high payment and low team satisfaction vs. low payment and high team satisfaction) and whether these profiles relate to different work-related outcomes such as job performance and work engagement. In line with this idea, researchers in the field of work motivation could identify individual motivation profiles by combing approaches from different motivational theories.

The fourth step of the LTA, the transition analysis, reveals whether profiles change over time. Because LTA is a person-centered approach that can investigate longitudinal phenomena, it sheds light on how individuals change over time instead of how relationships between two (or more) variables vary. Thus, LTA contributes significantly to many contexts where the focus is on changes in individuals. For instance, occupational health psychologists may examine changes in employees’ psychological health based on a combination of different health indicators. Moreover, as an alternative to traditional longitudinal methods for training evaluation, OP researchers (and practitioners) could investigate whether leadership training is responsible for a transition from one (negative) leadership profile to another (positive) leadership profile using LTA.

LTA can investigate covariables to examine the transition from one profile to another. This step helps gain further insights into variables related to the development and change of the found profiles. For instance, if researchers want to speed up the transition from one profile to another (e.g., from low to high health profile), finding covariates is important. Regarding our applied example, staying in the dominant high PsyCap profile may benefit individuals’ health, and this “no transition” is more likely when LMX is high than low. Thus, LTA revealed the importance of high LMX for high PsyCap.

### Key steps OP researchers can use when conducting LTA

When conducting an LTA, the main steps are estimating profiles, testing measurement invariance, defining and labeling the profiles, estimating transition probabilities, and adding covariates (Muthén and Muthén, 2000; Asparouhov and Muthén, 2014; Ryoo et al., 2018; Woo et al., 2018; Spurk et al., 2020). We have described each statistical step in detail; however, some other important steps must be discussed.

First, research should choose a meaningful set of variables for LTA. We chose the facets of PsyCap in our applied examples as the facets are theoretically connected. However, researchers may also be interested in profiles of variables from relegated constructs. In this case, we recommend choosing the set of variables to extract the profiles for LTA on a theoretical basis (Spurk et al., 2020).

Second, it is important to choose significant time frames when investigating the transition (Ryoo et al., 2018). Some profiles may change weekly, while other changes need more time to occur. In our applied example, we only find a few transitions between 6 weeks. One may speculate that because PsyCap is relatively stable, changes need more time to occur. Thus, the time frame may explain why transitions could not be identified.

Third, an important step for analyzing data using person-centered approaches is to handle outliers. As extreme outliers might affect the number of profiles attained and result in extreme profiles with only a few cases (Vermunt and Magidson, 2002), we encourage researchers to handle outliers before data analysis.

Fourth, covariables should be chosen based on a theoretical background (Ryoo et al., 2018; Spurk et al., 2020). We encourage research to provide a detailed explanation for the inclusion of covariates. Because identifying the number of profiles is exploratory, it might seem that the covariates are also based on exploration choice. However, we suggest that researchers should argue why a covariate affects the transition of a set of variables, even if the exact combination of the variables is unknown. For instance, in our applied example, we argued that LMX might relate to the transition probabilities because LMX is shown to be related to PsyCap mechanisms in general.

Finally, earlier research (Nylund-Gibson and Choi, 2018) recommended a sample size larger than 300 participants to get robust profiles in LTA. Based on this, our sample might be too small to conduct an LTA. However, because our primary aim was to present a five-step approach to conducting LTA using an illustrative example, future research should replicate our findings with larger samples. We aimed to demonstrate the usage of the steps and provide a practical example, regardless of the results in our applied example.

## Conclusion

We presented a step-by-step guide on conducting an LTA and provided the full Mplus syntax for researchers for free use online. By employing LTA, we conducted an applied example and found that PsyCap profiles are relatively stable across time (i.e., across two waves). In addition, we demonstrated a step-by-step approach for using LTA in organizational psychology. We provided a five-step approach and used our applied example to show how researchers can conduct LTA. By applying more LTA, researchers could better understand that changes in a set of variables over time (e.g., profile transitions) are sometimes more meaningful than changes in single variables.

## Data availability statement

The datasets presented in this study can be found in online repositories. The names of the repository/repositories and accession number(s) can be found at: https://osf.io/wdc4m/.

## Ethics statement

The studies involving human participants were reviewed and approved by the ethics committee of the University of Bamberg (dossier number 2021-02/04; 17 April 2021). The patients/participants provided their written informed consent to participate in this study.

## Author contributions

JZ and CB contributed to the conception and design of the study, conducted the statistical analyses, and wrote the first draft. SS and JZ wrote the discussion and were responsible for reviewing and editing the first draft. All authors contributed to the article and approved the submitted version.

## Conflict of interest

The authors declare that the research was conducted in the absence of any commercial or financial relationships that could be construed as a potential conflict of interest.

## Publisher’s note

All claims expressed in this article are solely those of the authors and do not necessarily represent those of their affiliated organizations, or those of the publisher, the editors and the reviewers. Any product that may be evaluated in this article, or claim that may be made by its manufacturer, is not guaranteed or endorsed by the publisher.

## Footnotes

^1^https://www.soscipanel.de (Accessed October 7, 2022).

^2^www.mindgarden.com (Accessed October 7, 2022).

^3^https://osf.io/wdc4m/?view_only=5a1aeb9c18404acfb0e471f7471dae1d (Accessed October 7, 2022).
